# Editorial: New knowledge of food microbiology in Asia, volume II

**DOI:** 10.3389/fmicb.2022.1061104

**Published:** 2022-10-31

**Authors:** Chunbao Li

**Affiliations:** College of Food Science and Technology, Nanjing Agricultural University, Nanjing, China

**Keywords:** food microbiology, sequencing, LAMP, antibiotic resistance, probiotic potential, Asia

In recent years, food microbiology has received widespread attention in Asia from many different directions. Several years ago, we collaborated with the journal *Frontiers in Microbiology* to launch a Research Topic New Knowledge of Food Microbiology in Asia, and got a good response from young scientists in this field. To continue in view of new developments, we relaunched the same Research Topic in November 2021.

In this volume, 17 manuscripts were submitted and all of them were strictly peer-reviewed. Finally, 14 manuscripts were accepted for publication. In research subjects ranged from Pseudomonas fluorescens and lactic acid in milk, Lactiplantibacillus plantarum in fermented foods, Escherichia coli and Proteus mirabilis in pigs and cattle, microbial contamination risk in pork production, general microbial community composition in different foods, and further. In these studies, some modern methodologies, e.g., high-throughput sequencing, were applied.

Mahata et al. characterized Sterigmatocystin produced by Aspergillus species from the Nidulantes Section in Foeniculum vulgare. Zhang, Lai et al. screened and evaluated lactic acid bacteria with probiotic potential from raw milk. Qu et al. sequenced Proteus mirabilis isolates recovered from pig farms and identified 95 virulence factors. Zhang, Bai et al. characterized Shiga toxin-producing Escherichia coli derived from a cattle farm. Yan et al. studied the persistent effects of acute exposure to AFB1 on rat liver and identified several regulatory factors, that is, genes Lama5, Gtse1, Fabp4, and Bcl6. Tao et al. analyzed the global prevalence of foodborne pathogens exhibiting antibiotic resistance and biofilm formation using meta-analysis. Sun et al. assessed the probiotic potentials of Lactiplantibacillus plantarum strains isolated from Chinese traditional fermented food through phenotypic and genomic analyses. Yang, Qiu et al. studied the dynamical changes of bacterial communities in Manila clam (Ruditapes philippinarum) during refrigerated storage. Du et al. tracked the complex community structures and regional variations of psychrotrophic bacteria in raw milk by single-molecule real-time sequencing and traditional cultivation techniques. Bai et al. made a genotyping diarrheagenic Escherichia coli based on CRISPR loci diversity and pathogenic potential. Yang, Zhao, et al. performed a risk analysis of microbial contamination risk in pork production using the quantitative exposure assessment model. Wang et al. evaluated chlorine tolerance and cross-resistance to antibiotics in poultry-associated Salmonella isolates in China, while Liu et al. studied the effect of bacterial resistance on Escherichia coli of in local large-scale pig farms. Bu et al. established a loop-mediated isothermal amplification (LAMP) assay for the rapid detection of Pseudomonas fluorescens in raw milk.

Taken together, we hope this volume provides some new insight into food microbiology in Asian countries.

## Author contributions

The author confirms being the sole contributor of this work and has approved it for publication.

## Conflict of interest

The author declares that the research was conducted in the absence of any commercial or financial relationships that could be construed as a potential conflict of interest.

## Publisher's note

All claims expressed in this article are solely those of the authors and do not necessarily represent those of their affiliated organizations, or those of the publisher, the editors and the reviewers. Any product that may be evaluated in this article, or claim that may be made by its manufacturer, is not guaranteed or endorsed by the publisher.

